# Supplementary material for: Gut microbiota composition of the isopod Ligia in South Korea exposed to expanded polystyrene pollution
Source: PLoS One. 2024 Aug 7;19(8):e0308246. doi: 10.1371/journal.pone.0308246 (PMC11305568; doi:10.1371/journal.pone.0308246)
Supplement: S1 Fig — The map image was created using a free, open-source base map program (the QGIS: QGIS is an open-source GIS tool) (Public Data Portal URL: https://www.data.go.kr/data/3035495/fileData.do) (Choi et al. [26]). (DOCX) [file pone.0308246.s001.docx]

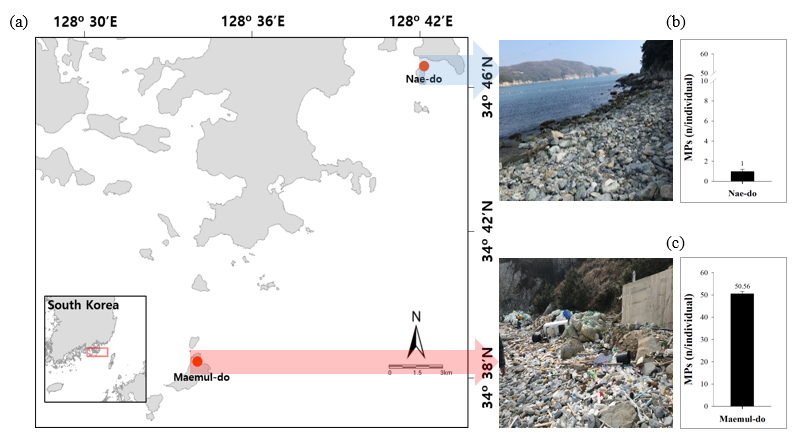


**S1 Fig**. (a) Two sampling locations of *Ligia* sp., and the number of microplastic (MP) contamination per individual organism (b) Nae-do (34°38’47.30”N 128°34’39.00”E), Geoje and (c) Maemul-do (34°47’21.50”N 128°42’58.10”E), Geoje. Map image was created using free open source basemap program (the QGIS:QGIS is open-source GIS tool) (Public Data Portal URL: https://www.data.go.kr/data/3035495/fileData.do) (Choi et al., 2023)
