# Supplementary material for: Gut microbiota composition of the isopod Ligia in South Korea exposed to expanded polystyrene pollution
Source: PLoS One. 2024 Aug 7;19(8):e0308246. doi: 10.1371/journal.pone.0308246 (PMC11305568; doi:10.1371/journal.pone.0308246)
Supplement: Attachment — Submitted filename: Responses to Reviewers_final.docx [file pone.0308246.s002.docx]

Responses to Reviewers’ Comments

Manuscript No.: PONE-D-24-19582

RESPONSES TO COMMENTS

We greatly appreciate the critical comments of the reviewers on our work. According to the reviewer’s comments, we thoroughly revised our manuscript and incorporated changes as suggested by the reviewer (please, check the red color in the revised manuscript). We answered one by one to comments raised by reviewers as follows:

Journal Requirements:

When submitting your revision, we need you to address these additional requirements.

1. Please ensure that your manuscript meets PLOS ONE's style requirements, including those for file naming.

Response: We confirmed that this revised manuscript meets PLOS ONE’s style requirements, such as file naming for supple figures, Titles, and affiliations according to the author’s guidelines.

2. In your Methods section, please provide additional information regarding the permits you obtained for the work. Please ensure you have included the full name of the authority that approved the field site access and, if no permits were required, a brief statement explaining why.

Response: We added the information regarding the permits of sampling locations in the method. “this area was an area where there was no permitting procedure required for marine *Ligia* spp. collection; thus, it was legal to freely sample. (Line 97-98)

3. We note that the grant information you provided in the ‘Funding Information’ and ‘Financial Disclosure’ sections do not match.

When you resubmit, please ensure that you provide the correct grant numbers for the awards you received for your study in the ‘Funding Information’ section.

Response: We removed “Funding information” from the revised manuscript and added a “financial disclosure” file.

4. Thank you for stating the following financial disclosure:

"This work was supported by the grants “Risk assessment to prepare standards for protecting marine ecosystem(KIMST-20230383)”and “Land and sea based input and fate of microplastics in the marine environment (KIMST-20230383)of the Korea Institute of Marine Science & Technology Promotion (KIMST) funded by the Ministry of Oceans and Fisheries”, and the National Research Foundation of Korea(NRF) grant funded by the Korea government(MSIT) (No. 2023R1A2C1005630)."

Please state what role the funders took in the study. If the funders had no role, please state: "The funders had no role in study design, data collection and analysis, decision to publish, or preparation of the manuscript." If this statement is not correct you must amend it as needed.

Please include this amended Role of Funder statement in your cover letter; we will change the online submission form on your behalf.

Response: We added the funder’s role in the financial disclosure file.

5. Please note that funding information should not appear in the Acknowledgments section or other areas of your manuscript. We will only publish funding information present in the Funding Statement section of the online submission form. Please remove any funding-related text from the manuscript.

Response: We removed the funding information in the revised manuscript.

6. Please provide a complete Data Availability Statement in the submission form, ensuring you include all necessary access information or a reason for why you are unable to make your data freely accessible. If your research concerns only data provided within your submission, please write "All data are in the manuscript and/or supporting information files" as your Data Availability Statement.

Response: We provide a complete Data Availability Statement in the revised manuscript.

"All data are in the manuscript and/or supporting information files" as your Data Availability Statement.” (Line 467)

7. PLOS requires an ORCID iD for the corresponding author in Editorial Manager on papers submitted after December 6th, 2016. Please ensure that you have an ORCID iD and that it is validated in Editorial Manager. To do this, go to ‘Update my Information’ (in the upper left-hand corner of the main menu), and click on the Fetch/Validate link next to the ORCID field. This will take you to the ORCID site and allow you to create a new iD or authenticate a pre-existing iD in Editorial Manager. Please see the following video for instructions on linking an ORCID iD to your Editorial Manager account: https://www.youtube.com/watch?v=_xcclfuvtxQ

Response: The corresponding author has been ORCID ID and checked its validation.

8. Please include your full ethics statement in the ‘Methods’ section of your manuscript file. In your statement, please include the full name of the IRB or ethics committee who approved or waived your study, as well as whether or not you obtained informed written or verbal consent. If consent was waived for your study, please include this information in your statement as well.

Response: Since arthropods, including *Ligia* spp., do not require permission from our institution's IRB committee, they have not been approved in this study. Tissues were separated by liquid nitrogen freezing immediately after field collection.

9. We note that Supporting Figure S1 in your submission contain map images which may be copyrighted. All PLOS content is published under the Creative Commons Attribution License (CC BY 4.0), which means that the manuscript, images, and Supporting Information files will be freely available online, and any third party is permitted to access, download, copy, distribute, and use these materials in any way, even commercially, with proper attribution. For these reasons, we cannot publish previously copyrighted maps or satellite images created using proprietary data, such as Google software (Google Maps, Street View, and Earth). For more information, see our copyright guidelines: http://journals.plos.org/plosone/s/licenses-and-copyright.

We require you to either (1) present written permission from the copyright holder to publish these figures specifically under the CC BY 4.0 license, or (2) remove the figures from your submission:

1) You may seek permission from the original copyright holder of Supporting Figure S1 to publish the content specifically under the CC BY 4.0 license.

We recommend that you contact the original copyright holder with the Content Permission Form (http://journals.plos.org/plosone/s/file?id=7c09/content-permission-form.pdf) and the following text:

“I request permission for the open-access journal PLOS ONE to publish XXX under the Creative Commons Attribution License (CCAL) CC BY 4.0 (http://creativecommons.org/licenses/by/4.0/). Please be aware that this license allows unrestricted use and distribution, even commercially, by third parties. Please reply and provide explicit written permission to publish XXX under a CC BY license and complete the attached form.”

Please upload the completed Content Permission Form or other proof of granted permissions as an ""Other"" file with your submission.

In the figure caption of the copyrighted figure, please include the following text: “Reprinted from [ref] under a CC BY license, with permission from [name of publisher], original copyright [original copyright year].”

2) If you are unable to obtain permission from the original copyright holder to publish these figures under the CC BY 4.0 license or if the copyright holder’s requirements are incompatible with the CC BY 4.0 license, please either i) remove the figure or ii) supply a replacement figure that complies with the CC BY 4.0 license. Please check copyright information on all replacement figures and update the figure caption with source information. If applicable, please specify in the figure caption text when a figure is similar but not identical to the original image and is therefore for illustrative purposes only.

The following resources for replacing copyrighted map figures may be helpful:

USGS National Map Viewer (public domain): http://viewer.nationalmap.gov/viewer/

The Gateway to Astronaut Photography of Earth (public domain): http://eol.jsc.nasa.gov/sseop/clickmap/

Maps at the CIA (public domain): https://www.cia.gov/library/publications/the-world-factbook/index.html and https://www.cia.gov/library/publications/cia-maps-publications/index.html

NASA Earth Observatory (public domain): http://earthobservatory.nasa.gov/

Landsat: http://landsat.visibleearth.nasa.gov/

USGS EROS (Earth Resources Observatory and Science (EROS) Center) (public domain): http://eros.usgs.gov/#

Natural Earth (public domain): http://www.naturalearthdata.com/

[Note: HTML markup is below. Please do not edit.]

Response: Our Supporting Figure S1 image was created using a free, open-source base map program (the QGIS: QGIS is an open-source GIS tool) (Public Data Portal URL: https://www.data.go.kr/data/3035495/fileData.do), and there is no copyright problem. We attached the copyright information of the map image in the revision and added website information on the legend of Figure S1 in the revised manuscript (Line 720-722)

|  |  |
| --- | --- |

[Information of the map image]

Reviewers' comments:

Reviewer's Responses to Questions

Comments to the Author

1. Is the manuscript technically sound, and do the data support the conclusions?

The manuscript must describe a technically sound piece of scientific research with data that supports the conclusions. Experiments must have been conducted rigorously, with appropriate controls, replication, and sample sizes. The conclusions must be drawn appropriately based on the data presented.

Reviewer #1: Yes

Reviewer #2: Partly

Reviewer #3: Partly

Response: We also checked the conclusion and corrected it based on the data.

2. Has the statistical analysis been performed appropriately and rigorously?

Reviewer #1: Yes

Reviewer #2: Yes

Reviewer #3: Yes

Response: We had appropriately performed the statistical analysis.

3. Have the authors made all data underlying the findings in their manuscript fully available?

The PLOS Data policy requires authors to make all data underlying the findings described in their manuscript fully available without restriction, with rare exception (please refer to the Data Availability Statement in the manuscript PDF file). The data should be provided as part of the manuscript or its supporting information, or deposited to a public repository. For example, in addition to summary statistics, the data points behind means, medians and variance measures should be available. If there are restrictions on publicly sharing data—e.g. participant privacy or use of data from a third party—those must be specified.

Reviewer #1: Yes

Reviewer #2: Yes

Reviewer #3: Yes

Response: Our manuscript included all of the data from the study.

4. Is the manuscript presented in an intelligible fashion and written in standard English?

PLOS ONE does not copyedit accepted manuscripts, so the language in submitted articles must be clear, correct, and unambiguous. Any typographical or grammatical errors should be corrected at revision, so please note any specific errors here.

Reviewer #1: Yes

Reviewer #2: Yes

Reviewer #3: No

Response: We also checked any typographical or grammatical errors in our manuscript, which were re-corrected from Cactus Communications Inc. (214 Carnegie Center, Suite 102, Princeton, NJ 08540, USA).

**5. Review Comments to the Author**

**Reviewer #1**: The manuscript is well written. The methodology used for identification, extraction and metagenomic sequencing of gut microbiota is well described with all the necessary details.

I would recommend adding the limitations of the metagenomic approach used the study in the conclusion section to make the manuscript well rounded.

Response: We added the limitations of the metagenomic approach and the requirement for further study in conclusion. (Line 458-462)

**Reviewer #2**:

Ln 73-77. Are there more evidences suggesting the involvement of isopod gut microbiome in plastic degradation?

Response: We additionally analyzed the publication database relating the isopod gut microbiome in plastic degradation but we could not find out the research report.

Ln99. How many samples were collected in total from the collection site? How many of these samples were utilized for further analysis and what was the criteria for choosing those samples?

Response: This study is a part of large-scale research “Effects of EPS on *Ligia* using multi-omics approach and isolation of plastic degrading bacteria from the gut of *Ligia*”. The parallel studies included plastic/chemical analysis, transcriptome analysis, and microbiome analysis. For this study, we used thirty wharf roaches from the total 100 individuals collected, but the sample size was reduced to ten individuals due to difficulties in the DNA extraction and screening process, such as body length (23.02 ± 1.17 mm) and body weight (0.70 ± 0.12 g) in order to minimize the difference between individuals (Lines 93-94). However, we tried to analyze more than 3 replicates at each site for statistical analysis. Since this study is a field study, sampling is always a challenge.

Ln 111. Has the same amount of template DNA been added into each of the reactions?

Response: Although it was not necessary to match the amount of DNA in equal quantities for amplification of the 16S rRNA gene, we used the 10 ng of DNA sample concentration for PCR analysis. We revised it (Line 108)

Ln121. How were the default parameters determined?

Response: The default parameters in GBLOCKs v.0.91b are as follows:

After alignment, ambiguous regions (i.e. containing gaps and/or poorly aligned) were removed with Gblocks (v0.91b) using the following parameters: minimum length of a block after gap cleaning: 5; positions with a gap in less than 50% of the sequences were selected in the final alignment if they were within an appropriate block; all segments with contiguous nonconserved positions bigger than 4 were rejected; and minimum number of sequences for a flank position: 55%

Ln 140. Have the authors used other RNA seq analysis tools to obtain more comprehensive data and avoid tool specific bias?

Response: Our study only covered results of mitochondrial 16S rRNA and metagenome shotgun sequencing analysis in the wharf roach gut. Therefore, we did not show any RNA-seq analysis data. In our previous manuscript, we showed the RNA-seq results in wharf roaches (Choi et al., 2023).

*Choi Y, Shin D, Hong CP, Shin DM, Cho SH, Kim SS, et al. The effects of environmental Microplastic on wharf roach (Ligia exotica): A Multi-Omics approach. Chemosphere 2023;335:139122.*

Ln 189. How was the quantity of plastic debris measured? How much area was used for measurement? Is the measurement consistent throughout the region and how is the isopod population affected by amount of debris found? Is it a linear correlation?

Response: In our previous results, we identified the MPs (by plastic size, type, and shape) using the micro-Fourier transform infrared microscope (μ Thermo Nicolet 6700 Continue μ FTIR; m; Thermo Scientific, Waltham, MA, USA). The 80% region of area was measured in our study. The number of particles was 1 particle in blank and reference site. The effect of population is not this research content, but the potential effects depend on microplastic contamination presented in our previous study (Choi et al., 2023). This study provides the first comparative field evidence of the gut microbiota dynamics of plastic detritus consumers in marine ecosystems.

*Choi Y, Shin D, Hong CP, Shin DM, Cho SH, Kim SS, et al. The effects of environmental Microplastic on wharf roach (Ligia exotica): A Multi-Omics approach. Chemosphere 2023;335:139122.*

**Reviewer #3**: The present manuscript assesses the role of Ligia sp. in plastic degradation and examines the potential impacts of microplastics on microbiome composition. This issue is of significant importance and interest, with considerable potential implications. However, several concerns must be addressed before publication:

Major Comments:

1) The authors describe "clean site" and "polluted site" for the collection of specimens in the materials and methods section. How did the authors characterize these sampling locations? This should be included in the materials and methods section. Considering that the authors have previously characterized the plastic debris in these areas in earlier studies, they should reference these studies in the materials and methods section.

Response: The location was classified according to the previous study that characterized the microplastic contamination of two sites (clean and polluted sites in our study) (Choi et al., 2023). This information was added in the revised manuscript (Lines 96-97).

*Choi Y, Shin D, Hong CP, Shin DM, Cho SH, Kim SS, et al. The effects of environmental Microplastic on wharf roach (Ligia exotica): A Multi-Omics approach. Chemosphere 2023;335:139122.*

2) The authors make important statements, such as in line 383: "Several reports have suggested that environmental microorganisms, including species within the genera Pseudomonas and Bacillus spp., can degrade plastics using plastic degradation enzymes with different efficiencies [63, 69]. These findings indicated that the gut microbiota of Ligia from MD may be more potent in plastic ingestion and degradation than that of Ligia from ND." The authors assume that the presence of certain taxonomic groups (at least at the genus level) indicates a higher potential to degrade plastics. Such statements, which are found throughout the manuscript, are highly speculative. The authors cannot assume that all members of the genus Vibrio or Bacillus participate in plastic degradation. It can be more acceptable if the authors increase the power of the 16s analysis and reach to species level, but they should point out that those species are known for having potential to degrade plastics, and if the enrichment of this species is due to higher amount of plastics in that environment would require further study.

Response: We added the limitation of this study and the requirement of further study at the conclusion part of the revised manuscript. (Lines 458-462)

3) Although some pathways from the microbiota are highlighted in figure 6, which is a key figure for this study, I strongly suggest that the authors perform a PICRUSt analysis to identify which metabolic pathways are enriched and associated with the microbiota. The results should be discussed alongside those in figure 6 to better understand other potential relationships withing the microbiota metabolism.

Response: Figure 6 focuses on the significant presence of bacteria with plastic-degrading enzymes in *Ligia*'s gut collected at polluted sites, not other pathways. Therefore, we used functional gene analysis using DEseq2 instead of PICRUSt analysis. This is related to our ongoing study on the isolation of plastic-degrading bacteria in *Ligia*'s gut. We added the bacterial species name in Figure 6 to show the association with the microbiota. Identification of other metabolic pathways is beyond our scope of study.

4) The biggest concern of this reviewer is the considerable variability that can be produced by multiple factors in this study, which should not be ignored. Throughout the manuscript, the authors emphasize the critical changes in the microbiome due to microplastics. However, different sampling areas, species, and conditions are used. In the conclusions, the authors state, "These findings suggest that the composition of the gut microbiome may be related to different habitats, environments, and diets, including the occurrence of plastic pollution." I strongly suggest that the authors revise the manuscript to focus on this conclusion instead. Considering this, the study loses strength. I suggest that the authors conduct experiments under controlled conditions, acclimatizing the Ligia and using a commercially available mix of microplastics and expanded polystyrene. Such results would clarify whether the observed changes are due to microplastics or other environmental conditions.

Response: I agree with the reviewer's comment. However, this study attempted to analyze the difference in the gut microbiome of microplastic consumers based on previous studies (Choi et al., 2023). Our conclusion is derived from the result that the genes related to plastic decomposition and related microorganisms (Fig 6) were significantly higher in the gut of *Ligia* collected from plastic-contaminated areas. Therefore, we also added the limitations and the need for further research (Lines 458-462). In addition, we are currently conducting a laboratory study to check whether there is a change in the intestinal microbiota of *Ligia* sp. by ingesting EPS within controlled environments. As suggested by the reviewer, our ongoing study will clarify whether plastic ingestion influences the changes in the gut microbiome in this species.

5) Similarly, I believe the study would benefit from additional in vitro experiments isolating the most interesting microbiota with potential to degrade microplastics and demonstrating this potential in vitro. If possible, metatranscriptome analysis should be conducted to identify which pathways change in the presence of these plastics.

Response: As the above answer, *in vitro* experiment is in progress, including the microorganisms with EPS resolution are additionally isolated, and resolution studies are being conducted by pure isolation of bacteria belonging to the genus *Pseudomonas*, *Sphingomonas*, *Bacillus*, etc. Additional long-term digestion experiment also includes metatranscriptome analysis.

Minor Comments:

1) Lines 72-74: I suggest introducing arthropods, such as insects, instead of directly discussing insects. This is confusing considering that the study model is a crustacean.

Response: As per the reviewer’s suggestion, we added the part about introducing arthropods in the discussion. (Line 66)

2) Have the authors assessed whether microplastics have any potential deleterious effects on any Ligia species? Or whether the presence of any Ligia species in non-contaminated areas and their absence in contaminated areas can be associated with microplastics?

Response: This study provides the key field evidence of the *Ligia species* gut microbiota dynamics of plastic detritus consumers in marine ecosystems. The microbiome plays critical roles in the training and development of major components of the host’s innate and adaptive immune system, while the immune system orchestrates the maintenance of key features of host-microbe symbiosis (Zheng et al. Interaction between microbiota and immunity in health and disease cell research 30,492-506, 2020). Therefore, adverse changes in the gut microbiome might be an important factor in immune and neuro dysfunction. Our previous research also addressed the potential impact of microplastic contamination in *Ligia* sp*.*
